# Self-Assembled Lipid Nanoparticles for Oral Delivery of Heparin-Coated Iron Oxide Nanoparticles for Theranostic Purposes

**DOI:** 10.3390/molecules22060963

**Published:** 2017-06-09

**Authors:** Eleonora Truzzi, Chiara Bongio, Francesca Sacchetti, Eleonora Maretti, Monica Montanari, Valentina Iannuccelli, Elena Vismara, Eliana Leo

**Affiliations:** 1 Department of Life Sciences, University of Modena and Reggio Emilia, via Campi 103, 41125 Modena, Italy; eleonora.truzzi@unimore.it (E.T.); francesca.sacchetti@unimore.it (F.S.); eleonora.maretti@unimore.it (E.M.); valentina.iannuccelli@unimore.it (V.I.); 2 Department of Chemistry, Materials and Chemical Engineering “G. Natta”, via Mancinelli 7, Politecnico di Milano, 20131 Milano, Italy; chiara.bongio@polimi.it; 3 Department of Life Sciences, University of Modena and Reggio Emilia, via Campi 287, 41125 Modena, Italy; monica.montanari@unimore.it

**Keywords:** theranostics, solid lipid nanoparticles, iron oxide nanoparticles, heparin coating, intestinal lymphatic absorption

## Abstract

Recently, solid lipid nanoparticles (SLNs) have attracted increasing attention owing to their potential as an oral delivery system, promoting intestinal absorption in the lymphatic circulation which plays a role in disseminating metastatic cancer cells and infectious agents throughout the body. SLN features can be exploited for the oral delivery of theranostics. Therefore, the aim of this work was to design and characterise self-assembled lipid nanoparticles (SALNs) to encapsulate and stabilise iron oxide nanoparticles non-covalently coated with heparin (Fe@hepa) as a model of a theranostic tool. SALNs were characterised for physico-chemical properties (particle size, surface charge, encapsulation efficiency, in vitro stability, and heparin leakage), as well as in vitro cytotoxicity by methyl thiazole tetrazolium (MTT) assay and cell internalisation in CaCo-2, a cell line model used as an indirect indication of intestinal lymphatic absorption. SALNs of about 180 nm, which are stable in suspension and have a high encapsulation efficiency (>90%) were obtained. SALNs were able to stabilise the heparin coating of Fe@hepa, which are typically unstable in physiological environments. Moreover, SALNs–Fe@hepa showed no cytotoxicity, although their ability to be internalised into CaCo-2 cells was highlighted by confocal microscopy analysis. Therefore, the results indicated that SALNs can be considered as a promising tool to orally deliver theranostic Fe@hepa into the lymphatic circulation, although further in vivo studies are needed to comprehend further potential applications.

## 1. Introduction

Currently, oral delivery is the most accepted route of drug administration, even though it is associated with poor drug bioavailability. One of the most promising strategies to overcome these limitations is the use of nanomedicine or nano-drug delivery systems [1]. As an example, solid lipid nanoparticles (SLNs) have attracted increasing attention owing to their biocompatibility and biodegradability. SLNs are composed of lipids in a solid state at room temperature and surfactants. They are produced using hot or cold homogenisation without the employment of organic solvents and generally have low production costs. SLNs offer advantages such as good tolerability, high oral drug bioavailability and low acute and chronic toxicity [2,3]. Moreover, being composed of lipids, SLNs have shown good potential in achieving drug delivery into the systemic circulation through intestinal lymphatic absorption [4,5,6]. After oral administration, small and hydrophilic substances enter in the systemic circulation by a passive absorption mechanism through enterocytes. On the contrary, large and lipophilic compounds with a logP ≥ 5 (where P is the octanol/water partition coefficient), such as components of SLNs, are metabolically stable (in the intestinal lumen and within enterocytes) and can be considered good candidates for lymphatic transport to the systemic circulation [7]. Drug adsorption via the intestinal lymphatic system has several major advantages, including circumventing first-pass metabolism and targeting drugs to diseases that spread through the lymphatic system. For example, cancer cells use the lymph nodes as a reservoir to spread to the other areas of the body [8].

The main ways to deliver drugs to intestinal lymphatic vessels are through lymphatic capillaries, gut-associated lymphoid follicles that form Peyer’s patch, and finally the intestinal walls via transcellular absorption. This last route is the lymphatic target of lipid-based nanoformulations because during transit across the enterocyte the lipids become associated with chylomicrons which are secreted into the mesenteric lymph duct [9,10,11,12].

SLNs have to satisfy certain requirements to achieve lymphatic delivery. It was observed that the uptake and fate of SLNs are influenced by particle size, surface hydrophobicity, type of lipids, and concentration of the emulsifier [1,6,13]. Also, the surface charge plays an important role: negatively-charged carriers have been reported to show higher lymphatic uptake than neutral or positively-charged particles [1,9,14]. SLNs promote lymphatic absorption and can also be exploited for theranostic purposes, which to the best of our knowledge, have not been extensively investigated [15]. Theranostics is the fusion of therapeutic and diagnostic approaches aiming to personalise and advance medicine. Magnetic nanoparticles (MNPs) represent a particularly appropriate tool based on their ability to be simultaneously functionalised and guided by external magnetic fields [16]. Some MNPs-based therapeutic applications include magnetic fluid hyperthermia (MFH), magnetic resonance imaging (MRI) and magnetic drug targeting [17,18]. In this field, iron oxide (Fe_3_O_4_) MNPs provide a unique nanoplatform with tunable sizes and surface chemistry studied extensively for MRI and MFH applications [19]. Without a coating, MNPs have hydrophobic surfaces with a high area to volume ratio and a propensity to agglomerate. An appropriate surface coating allows MNPs to be and remain homogenously dispersed for longer times. Several materials have been used to modify the surface of MNPs, such as organic polymers (dextran, chitosan, polyethylene glycol), organic surfactants (sodium oleate and dodecylamine), and metals [16]. Vismara et al. proposed the use of heparin as a non-covalent coating for iron oxide nanoparticles (Fe@hepa) [20]. Heparin, a natural polysaccharide with many bioactive properties, is a heterogeneous, polydispersed, highly sulphated glycosaminoglycan composed of 1 → 4 linked disaccharide repeating units. Each unit consists of an α-d-glucosamine and either a hexuronic acid, α-l-idruronic or β-d-glucoronicacid unit, with *O*-sulphate groups at different positions of the disaccharide. Various studies have demonstrated that heparin and low-molecular-weight heparins, in addition to having anticoagulant properties, are anti-angiogenic agents and can be used as vectors to reach tumour sites due to their ability to bind over-expressed proteins [21,22,23]. Thanks to these features, the heparin coating specifically directs iron oxide nanoparticles to tumour environments in order to accomplish the theranostic aim. Moreover, Vismara et al. demonstrated an increased stability in a water suspension of Fe@hepa nanoparticles with respect to naked iron oxide by conferring a negative charge due to the heparin coating [20]. However, the heparin surface shell is instable in physiologic environment where the presence of ions reduces the strength of the electrostatic bond between the positive iron oxide core and the negative heparin chain.

Therefore, the purpose of the present work was to design a nano-theranostic tool based on Fe@hepa nanoparticles for oral absorption through the lymphatic route. To the best of our knowledge, in the theranostic field poor attention has been addressed to the study of this promising approach. In order to stabilise the heparin coating in physiological environments, and at the same time promote oral absorption through the lymphatic route, Fe@hepa were encapsulated in a biocompatible solid lipid shell to obtain self-assembled lipid nanoparticles (SALNs). SALNs were obtained by self-emulsification process and were characterised with regard to their size, encapsulation efficiency, in vitro cytotoxicity and ability to be internalised into the CaCo-2 cell line (colon rectal adenocarcinoma cell line of human origin) used as a model for an indirect indication of lymphatic uptake.

## 2. Results

### 2.1. SALN Characterisation

By using the original self-emulsification process, two SALNs–Fe@hepa samples were developed using 1 or 5 mg of Fe@hepa (namely SALNs–Fe@hepa1 and SALNs–Fe@hepa5, respectively). The particle size, polydispersity index (PDI) and Z-potential values obtained with photon correlation spectroscopy (PCS) analysis are shown in Table 1. No differences in the particle size nor in the PDI values were observed, regardless of the amount of Fe@hepa used (all the samples were roughly of 180 nm with a PDI of 0.3), while the negative charge of the particle surface (Z-potential value) increased with the increase of the initial amount of Fe@hepa utilised in the preparation. The particle size was monitored for one month and no significant changes were observed (data not shown). The size and the Z-potential of naked Fe@hepa were previously reported [20] and were 92 nm and −61 mV, respectively.

### 2.2. Morphological Studies

**Figure 1:**
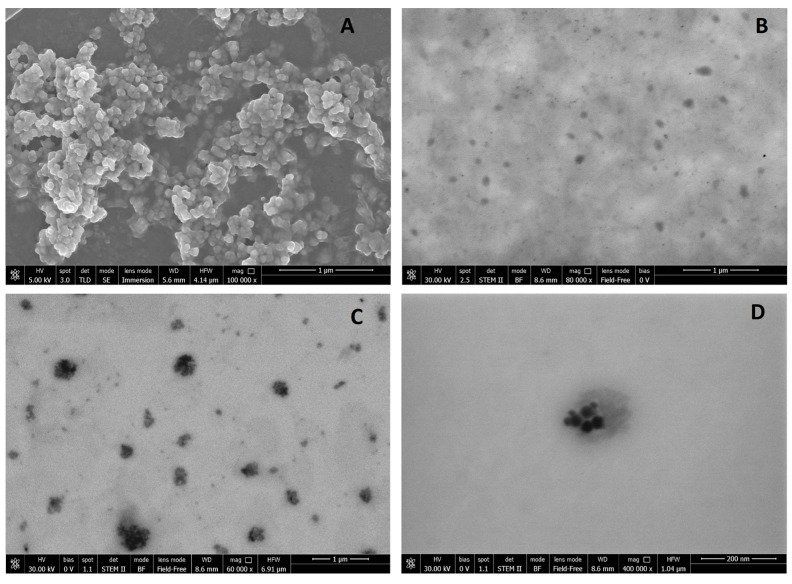
(**A**) SEM microphotograph of SALNs–Fe@hepa1 at high magnification (100,000×); (**B**) Representative scanning transmission electron microscopy (STEM) image of unloaded SALNs; (**C**) Representative STEM image of SALNs–Fe@hepa5 at low magnification (60,000×); (**D**) Representative STEM image of SALNs–Fe@hepa5 at high magnification (400,000×).

Morphological characterisation of the samples was performed using the scanning electron microscopy analysis (SEM modality) to visualise the particles in solid form, while the scanning transmission electron microscopy analysis (STEM modality) was used to observe the samples as suspension. Both the analyses were performed in high-vacuum conditions. Figure 1A shows, as an example, the image of SALNs–Fe@hepa1 at high magnification (100,000×) using the SEM technique. Even if SALNs appear aggregated in clusters, each single particle can be clearly recognised as a distinct solid structure with a roughly spherical morphology.

By STEM modality (Figure 1B), unloaded SALNs in suspension are hardly detectable due to their intrinsically low electron density that limits the resolution. However, even if the particles appear as weak-contrast dark formations, their imperfectly spherical morphology is easily observable.

Figure 1C,D shows the STEM images of loaded particles (SALNs–Fe@hepa5). At low magnification (Figure 1C), SALNs–Fe@hepa5 appear irregular in the shape, as observed also for the unloaded particles, but with a darker inner structure. The high-contrast dark part in the core region of each particle can be assigned to the Fe@hepa clusters, while the clear part surrounding the core regions can be attributable to the lipid shell. At high magnification (Figure 1D) one single particle with a rough contour is observed. Within the particle, even if not perfectly in the centre, dark small dots, due to clusters of Fe@hepa nanoparticles, are clearly visible. The clusters appear surrounded by a weak-contrast dark part attributable to the lipid matrix, according to the images of the unloaded sample (Figure 1B). The particle sizes of SALNs–Fe@hepa5 as well as of the Fe@hepa nanoparticles are also consistent with PCS analysis results reported in Table 1 and in previous studies [20], respectively.

**Figure 2:**
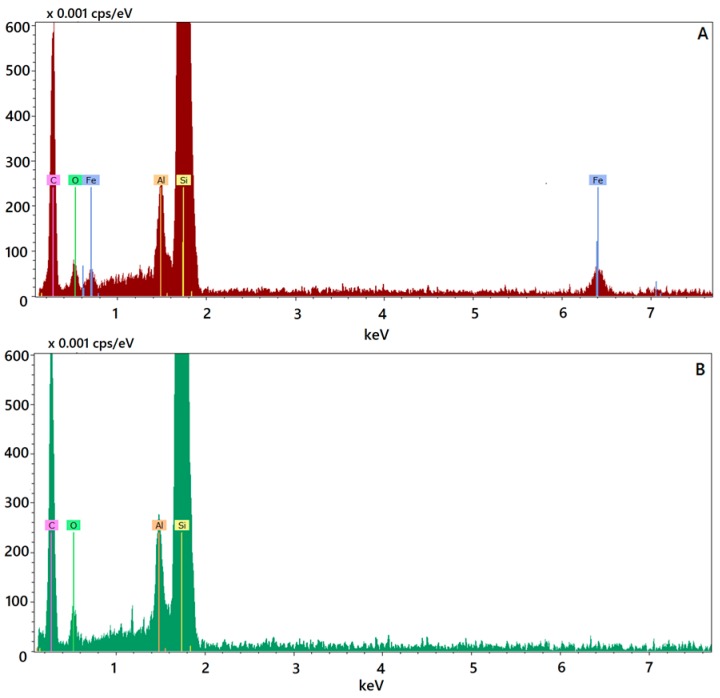
Energy dispersive X-ray (EDX) spectra referred to SALNs–Fe@hepa5 (**A**) and unloaded SALNs (**B**).

To confirm the composition of the particles observed by the electron microscopy analysis, the qualitative energy dispersive X-ray (EDX) analysis was performed by the single-point method. A single loaded nanoparticle, as observed in the image reported in Figure 1D, was analysed in comparison with an individual unloaded nanoparticle and the qualitative composition was reported in the EDX spectra representing the plots of X-ray counts vs. elements. In spectrum relating to SALNs–Fe@hepa, the peak of iron is clearly visible (Figure 2A), while it is absent in unloaded particles (Figure 2B), confirming the presence of Fe@hepa into the loaded SALNs. In the spectra, the presence of Al and Si are probably due to the support used for the analyses.

### 2.3. Drug Loading and Encapsulation Efficiency

The amount of Fe@hepa loaded inside SALNs was calculated by indirect method, i.e., analysing the non-encapsulated amount of Fe@hepa. No significant differences are observed in the encapsulation efficiency (EE%) between the samples (*p* > 0.05), while the drug loading (DL) increases five-fold in SALNs-Fe@hepa5 (*p* < 0.001) (Table 2).

### 2.4. In Vitro SALNs-Fe@hepa Stability

**Figure 3:**
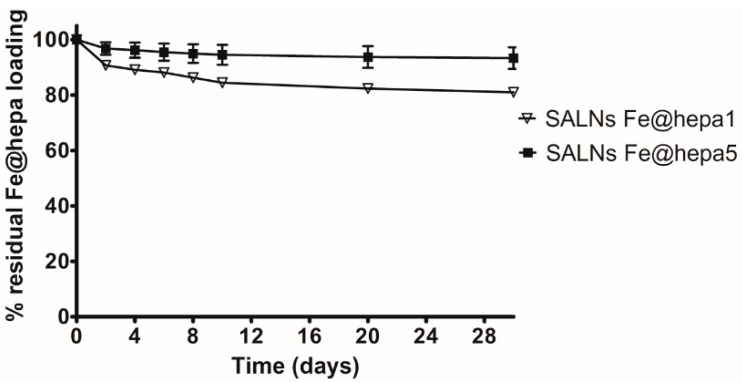
Percentage of Fe@hepa released from SALNs-Fe@hepa1 and SALNs–Fe@hepa5 stored as a suspension at 4 °C for one month, where 100% corresponds to initial SALNs–Fe@hepa drug loading. Error bars indicate standard deviation (SD); where not visible, error bars did not exceed symbol size.

In order to verify the retention of Fe@hepa in SALNs stored as suspension at 4 °C, the spontaneous Fe@hepa sedimentation from SALNs–Fe@hepa was monitored for both the samples for one month after the preparation (t_0_). At predetermined time interval, the amount of Fe@hepa separated from the suspension was measured by spectrophotometric method. These data were subtracted from the initial Fe@hepa loading and the results are reported in the graph (Figure 3). The data indicate that SALNs–Fe@hepa5 are more stable compared to the SALNs–Fe@hepa1. Indeed, for this sample, after 4 days, the loss of the cargo was only 4% compared to the initial content, indicating a good stability of the system. Then, a very slow sedimentation rate of free Fe@hepa is observed in the remaining time until a total loss of 6%. On the contrary, SALNs–Fe@hepa1 appeared quite instable in suspension, showing a fast initial loss of cargo corresponding to about 11% in 4 days followed by a slower phase of Fe@hepa release up to a total loss of 20% in one month. Therefore, given the weak stability in suspension of SALNs–Fe@hepa1, this sample was not taken into account in the further experiments.

### 2.5. Stabilisation of Heparin Coating

In order to evaluate if the encapsulation of the Fe@hepa into SALNs was able to stabilise the heparin coating, the leakage of heparin form both naked Fe@hepa and SALNs-Fe@hepa5 was measured in physiologic solution (NaCl 0.9%). Indeed, the heparin shell is stable in water [20] but in the presence of saline medium the interaction between heparin and iron oxide became weaker, resulting in the release of heparin and in the loss of stability of the colloidal suspension [24]. Therefore, to measure the stability of the coating, experimental conditions with minimal perturbation (saline solution) were considered and the amount of heparin released after only 1 h at room temperature was evaluated. As reported in Table 3, in the case of naked Fe@hepa, as expected, a leakage of about 70% of the initial amount of heparin occurred while in the case of the SALNs–Fe@hepa5 no release of heparin in solution was observed in the time period considered.

### 2.6. Cytotoxicity Assay

**Figure 4:**
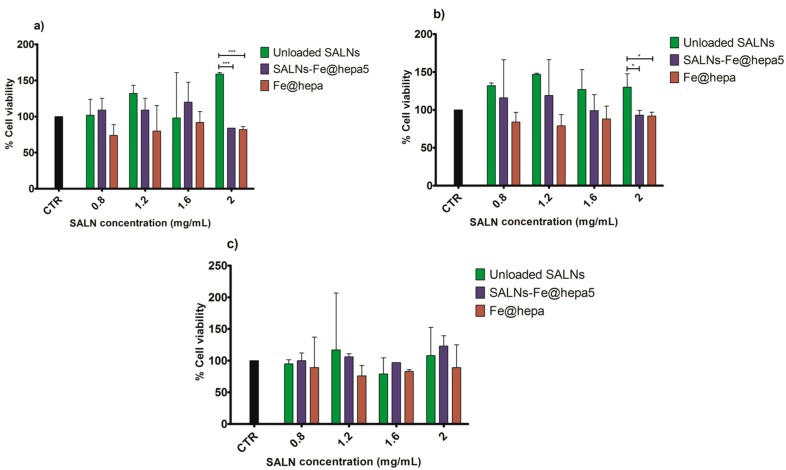
Analyses of cytotoxicity of the samples at different concentrations on CaCo-2 cells (colon rectal adenocarcinoma cell line of human origin) after 2 (**a**), 4 (**b**) and 6 h (**c**) of treatment, using methyl thiazole tetrazolium test (MTT) assay. SALN concentrations of 0.8, 1.2, 1.6, 2 mg/mL correspond to 21, 32, 42, 53 µg/mL of naked Fe@hepa, respectively. Comparison between samples was performed by ANOVA one-way test. Statistical significance levels were defined as: * (*p* < 0.05), *** (*p* < 0.001). Error bars indicate SD; where not visible, error bars did not exceed symbol size.

In order to determine the in vitro cytotoxicity of SALNs–Fe@hepa5 compared to naked Fe@hepa and unloaded SALNs, the methyl thiazole tetrazolium test (MTT) was performed on the intestinal CaCo-2 cell line after different incubation times (2, 4, and 6 h). For each time, various concentrations of SALNs (0.8, 1.2, 1.6, 2 mg/mL) and the respective amount of Fe@hepa (21, 32, 42, 53 µg/mL) were tested and the results are reported in Figure 4. The cytotoxicity of Fe@hepa is always higher than that of the other samples, but the cellular viability never dropped below 74%. Unloaded SALNs show a higher cell viability with respect to the control, while SALNs–Fe@hepa5 show a cell viability intermediate between the other two samples. However, significant differences are evident only within unloaded SALNs and the other two samples at the concentration of 2 mg/mL after 2 and 4 h of treatment.

### 2.7. Quantification of SALNs–Fe@hepa in the CaCo-2 Cell Line

Considering the results obtained from the study of cytotoxicity, the concentration of SALNs equal to 2 mg/mL (corresponding to 53 µg/mL of Fe@hepa) is considered optimal to study the internalisation of the systems in the CaCo-2 cell line, a colorectal cell line adopted as a model for lymphatic absorption [10,25].

Cells were preventively incubated for 2, 4 and 6 h with naked Fe@hepa and SALNs–Fe@hepa5, washed with phosphate buffer saline (PBS) and then lysated.

**Figure 5:**
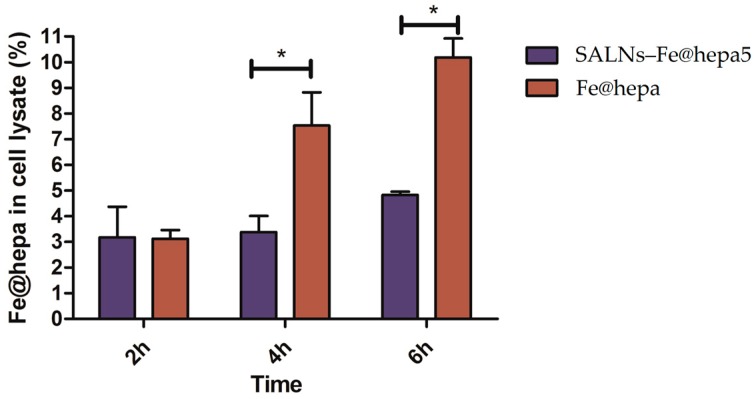
Quantification of iron oxide in CaCo-2 cells after 2, 4 and 6 h of treatment with SALNs–Fe@hepa5 at the concentration of 2 mg/mL (corresponding to 53 µg/mL of Fe@hepa) and naked Fe@hepa at the concentration of 53 µg/mL. A comparison between samples was performed by ANOVA one-way test. Statistical significance levels were defined as: * (*p* < 0.05).

The percentages of iron oxide found in the cell lysate respect to the amount used for the incubation are shown in Figure 5. After two hours of incubation, the percentage of iron oxide present into cell lysate was the same for both the samples (naked Fe@hepa and SALNs–Fe@hepa5). For the other incubation times, iron oxide in cell lysate resulted higher after the treatment with naked Fe@hepa. Moreover, a time-dependent correlation can be noticed: the amount of iron oxide increases with the increasing incubation time. Significant differences can be observed between the samples after 4 and 6 h of incubation (*p* < 0.05).

**Figure 6:**
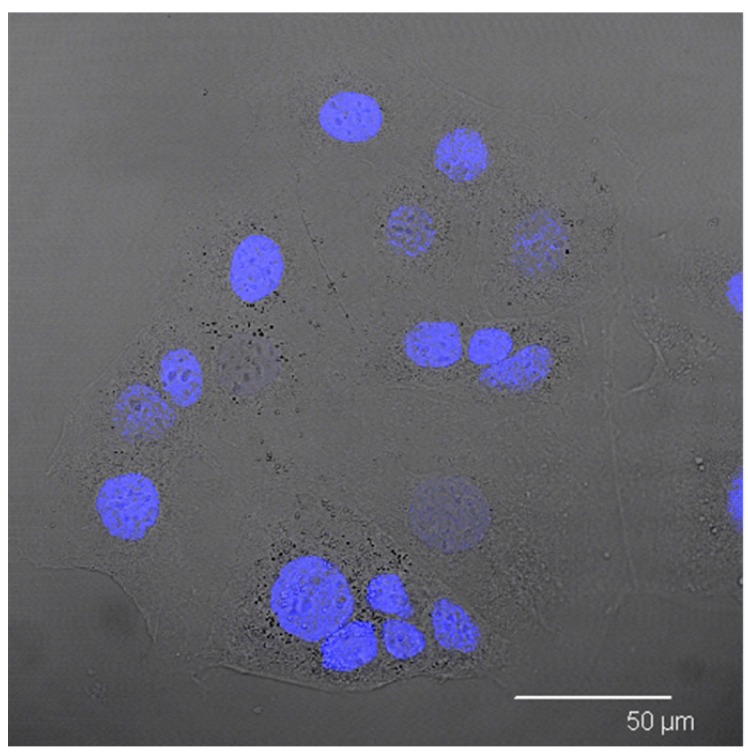
Confocal microscopy image of CaCo-2 cells after nuclei staining with Hoechst.

In order to visualise the internalisation of the sample in the CaCo-2 cell model (Figure 6), confocal laser scanning microscopy analysis was performed. Cellular nuclei were stained in blue, SALNs were labelled in red, while Fe@hepa, owing to their density, were visible as black spots in white-light channel.

**Figure 7:**
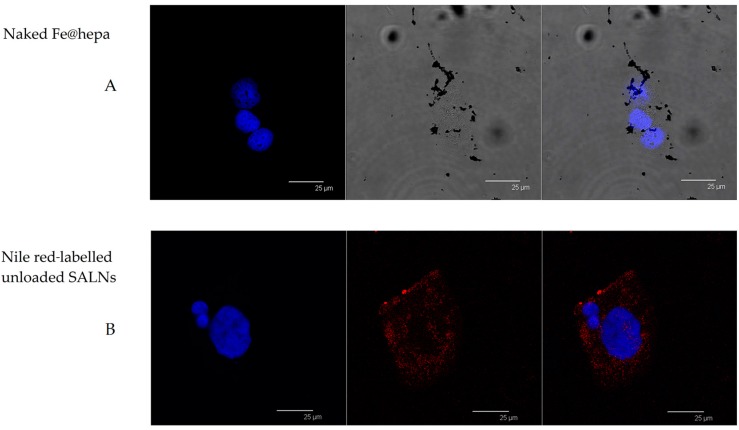
(**A**) Confocal microscopy images of CaCo-2 cells after nuclei staining and 4 h incubation with naked Fe@hepa. From left to right: blue channel (Hoechst), white-light channel and merged image. (**B**) Confocal microscopy images of CaCo-2 cells after nuclei staining and 4 h of incubation with Nile red-labelled unloaded SALNs. From left to right: blue channel (Hoechst), red channel (Nile red) and merged image.

CaCo-2 cells were treated with Nile red-labelled unloaded SALNs, Nile red-labelled SALNs–Fe@hepa5 and naked Fe@hepa. Figure 7A shows CaCo-2 cells incubated with Fe@hepa. Black spots with different sizes, attributed to clusters of iron oxide nanoparticles, are visible near the cytoplasm but clearly in a different z-thickness compared to the nuclei.

After incubation with Nile red-labelled unloaded SALNs (Figure 7B), red fluorescence is noticeable around the nuclei of CaCo-2 cells. Red spots are attributable to Nile red-labelled SALNs because no red fluorescence is observed in untreated CaCo-2 cells (Figure 6). The image clearly indicates that SALNs are localised inside the cells because the red fluorescence is located in the area surrounding the nuclei correspondent to the cytoplasm.

**Figure 8:**
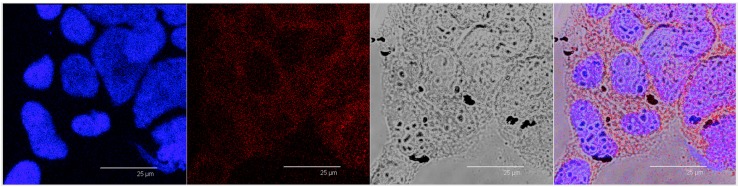
Confocal microscopy images of CaCo-2 cells after nuclei staining and 4 h of incubation with Nile red-labelled SALNs-Fe@hepa5. From left to right: blue channel (Hoechst), red channel (Nile red), white-light channel and merged image.

After treatment with Nile red-labelled SALNs-Fe@hepa5 (Figure 8), in addition to red spots, it is possible to appreciate, in a white-light channel, a grey shading around the nuclei where also red fluorescence is located. Moreover, black spots attributable to Fe@hepa clusters are clearly visible externally to cells revealing the presence of non-encapsulated Fe@hepa, which have tendency to form clusters outside the cells.

## 3. Discussion

Iron oxide nanoparticles have attracted considerable interest due to their superparamagnetic properties and their potential biomedical applications. The dimensions of these nanoparticles make them ideal candidates for nano-engineering of surfaces to develop non-toxic and biocompatible nanoparticles. Moreover, different coating materials can prevent their irreversible aggregation in aqueous or biological media [26]. Vismara et al. proposed heparin coating as an attractive strategy to achieve a theranostic aim exploiting anti-angiogenic activity of native heparin [20]. However, this coating is instable in physiological conditions. The goal of this work was to stabilise Fe@hepa by encapsulation in SALNs, envisaging an oral absorption through a lymphatic route.

SALNs are biocompatible, biodegradable, and can be used as controlled drug delivery and targeting system. Owing to their composition, SALNs possess a structure very similar to that of glyceride-rich chylomicrons which are believed to allow lymphatic transport of drugs into the intestinal lymphatic circulation [27].

It is known that lipid nanoparticles based on triglycerides with a high carbon chain length are less susceptible to intestinal lipase than those composed of a shorter carbon chain and are preferably transported into the intestinal lymphatic system [11,25,28]. For this reason, Geleol™ and Gelucire 50/13^®^ high carbon chain length lipids, both generally recognised as safe and biocompatible materials (manufacturer’s information), were selected as the lipid matrix for the SALN preparation. Moreover, these lipids have a low melting point, avoiding the risk of degradation of the drug during the preparation. Gelucire 50/13^®^ is composed of mono-, di-, and triglycerides with mono- and di-fatty acid esters of polyethylene glycol (PEG). Owing to the composition, it exhibits surfactant and solubility enhancing properties that can be exploited to better incorporate lipophilic compounds and to stabilise the lipid nanosystem [29,30]. Moreover, it has been demonstrated that Gelucire^®^ decreases P-glycoprotein efflux, making it a good candidate to gain lymphatic uptake [31,32].

After different formulation attempts, the preparation was optimised to achieve a reproducible and stable colloidal suspension without an observed particle dimensional change when Fe@hepa were encapsulated in the lipid matrix. The average diameter (around 180 nm) and the lipid nature of the particles make this system potentially suitable for intestinal lymphatic uptake associated with chylomicrons synthesised within enterocytes [1,6]. Alternatively, large molecular weight drug-carrier constructs may be selectively taken up intact via the lymphatic system because their large size favours uptake via the leakier structure of the lymphatic vessels, as compared to blood capillaries [33]. The size of the particles suitable for this pathway is a controversial matter [34]. However, it is recognised that the minute size of this formulation enables efficient uptake into the intestine, particularly via the lymphatic route, favoured by particles between 20 and 500 nm in diameter [9].

Regarding the zeta potential, unloaded SALNs measured slightly negative (−16 mV), and became progressively more negative with increasing amounts of Fe@hepa. Considering that Fe@hepa are strongly negative (about −61 mV) due to the presence of heparin coating, the more negative surface charge observed for SALNs–Fe@hepa with respect to unloaded SALNs can be attributable to a portion of Fe@hepa next to the SALN surface as observed in the STEM pictures (Figure 1C,D).

Morphological studies were performed to better understand the nanoparticle structure. The images obtained by SEM modality on the dried samples confirmed that the structure of the system is solid due to the lipid core made of solid components (Gelucire 50/13^®^ and Geleol™), as can be seen in Figure 1A. However, the SEM modality does not allow the observation of particle contour due to the poor resolution under low-voltage operating conditions (5 kV). Thus, pictures of SALNs in suspension were obtained in STEM modality, highlighting a rough surface structure probably due to the presence of a mixture of the two lipids in the particle matrix. In Figure 1D, at high magnification, it is possible to observe black spots, attributable to Fe@hepa. The clusters of Fe@hepa appear surrounded by the lipid matrix and the presence of Fe@hepa nanoparticles located in a peripheral position in the SALNs are also visible (Figure 1C,D). The presence of Fe@hepa close to the surface of the particles might explain the negative Z-potential value noticed for SALNs–Fe@hepa5. In addition, this finding is in agreement with EDX analysis that shows the superficial elemental composition of the particles. Indeed, EDX study shows the clear presence of iron in the spectrum of SALNs-Fe@hepa (Figure 2A) while no iron signal is evident in the spectrum of unloaded SALNs. On the other hand, the incorporation of Fe@hepa into the lipid matrix, as observed by electronic microscopy, should lead to a stabilisation of the heparin coating. To confirm if this goal was achieved, the release of heparin from the system in physiologic solution was evaluated comparing naked Fe@hepa and SALNs–Fe@hepa. The results (Table 3) show that no heparin was released from the SALNs–Fe@hepa sample, indicating that the encapsulation of Fe@hepa inside SALNs is a good strategy to avoid the loss of heparin coating occurring for the naked Fe@hepa. Indeed, heparin, selected as an iron oxide coating for its antiangiogenic features in tumour environments [21,22,23], is linked to the particle surface by ionic bonds between the positive iron oxide core and its negative chain. Therefore, Fe@hepa are destabilised in biological isotonic fluid where the electrostatic interaction between iron oxide and heparin become weaker. On the other hand, when Fe@hepa are surrounded by lipid matrix, the interaction with the biological fluids is avoided and no leakage of heparin occurs.

To better understand the potential of Fe@hepa–loaded SALNs, the particles were prepared using two different dosages (1 or 5 mg). The analyses indicate that in both SALNs–Fe@hepa samples the percentage of Fe@hepa incorporated is around 90%. This means that, increasing the initial loading, the encapsulation efficiency remains stable, suggesting that using only 1 mg of Fe@hepa the loading capacity of the lipid system was far from saturation. As evidence of this, increasing the initial amount of drug by five-fold, the loading increases proportionally from 5.14 µg/mg to 26.4 µg/mg (Table 2). However, during storage it was possible to notice a progressive sediment of Fe@hepa, indicating a probable desorption of Fe@hepa nanoparticles from the system. For this reason, the stability of SALNs–Fe@hepa samples was monitored for one month after the preparation (Figure 3). The data indicated that during one month, the higher loaded sample (SALNs–Fe@hepa5) was by far more stable than the less loaded sample (SALNs–Fe@hepa1). It can be assumed that in both the cases, the initial rapid loss of cargo is probably due to Fe@hepa non-embedded inside the lipid matrix or highly dispersed in the suspension. Afterwards, a release of Fe@hepa with a slower rate was observed; this was attributed to a leakage of Fe@hepa owing to its high density and to the magnetic forces between iron oxide nanoparticles. The results indicate clearly that the loss of Fe@hepa was larger for the lower loaded sample (SALNs–Fe@hepa1). To explain this finding, it can be assumed that when high amounts of Fe@hepa are embedded in the lipid matrix, the forces of attraction within Fe@hepa clusters are prevalent, stabilising the cargo. On the contrary, when poor amounts of Fe@hepa are loaded, the forces of attraction of the clusters inside the particles are weaker in respect to the attraction of the non-embedded particles, leading to a progressive leakage of the cargo. For this reason, all subsequent studies on cells were conducted using only the most loaded and stable sample (SALNs–Fe@hepa5).

The MTT assay on CaCo-2 cells was performed after different times of exposure (2, 4, 6 h) to compare the cytotoxicity induced by unloaded SALNs, naked Fe@hepa, and SALNs–Fe@hepa5. The CaCo-2 cell line was used because it has been reported to be an indirect indication of intestinal lymphatic transport [10,25]. The results of the analyses indicated that the lipids used to develop SALNs are not toxic but, on the contrary, they seem to improve the cell viability as the percentage of cell vitality observed after the treatment with unloaded SALNs resulted equal to or higher than the control (Figure 4). Only a slight cytotoxicity was observed for naked Fe@hepa since cell viability, at the experimental conditions adopted, never dropped below 74% compared to the control. Cytotoxicity studies reported in the literature and conducted on naked iron oxide nanoparticles demonstrated that these systems induce a reduction of cell viability depending on their coating, time of exposure, concentrations and cell type evaluated [35,36,37]. Thus, the results obtained in this work demonstrated that the coating with heparin allows biocompatible and non-toxic nanoparticles to be obtained. The cytotoxicity of SALNs–Fe@hepa falls in the middle between that of unloaded SALNs and Fe@hepa at all concentrations and incubation times considered, probably because the partial negative effects of Fe@hepa are compensated by the positive effect of unloaded SALNs. Therefore, it is possible to conclude that all the samples, at all the concentrations tested, do not exhibit toxicity on CaCo-2 cell model and the results indicate that the cytotoxicity is neither time- nor concentration-dependent. For this reason, to carry out the studies regarding the ability of the particles to enter the CaCo2 cells, the highest concentration (cell viability more than 80%) has been selected and therefore all the experiments were conducted using the concentration of 2 mg/mL.

The ability of the particles to enter in CaCo-2 cells was evaluated by measuring the amount of iron transported in the cells by the two systems (Fe@hepa and SALNs-Fe@hepa5). The results (Figure 5) indicated that the amount of iron found in the cells was higher for the cells incubated with naked Fe@hepa respect to cells incubated with SALNs–Fe@hepa, especially for longer incubation times. These findings seem to be in contrary to expectations because in the literature SALNs resulted able to improve the internalisation of drugs thanks to their composition [4,5,6]. However, it is important to notice that the higher percentage of iron found in the cells treated with Fe@hepa might be due to the precipitation of naked iron oxide on the bottom of the wells, because of the loss of the heparin coating in biological fluids. Indeed, during the experiments, it was observed that in the case of cells treated with naked Fe@hepa, dark spots attributable to iron remained attached to the well bottom, even after the washing with PBS (see Section 4.11). On the contrary, in the case of cells incubated with SALNs–Fe@hepa5, the non-internalised particles were easily removed with washing owing to the low density of their lipid composition. As a result, in the case of cells treated with Fe@hepa an overestimation of Fe@hepa associated with the cells might have occurred.

In order to support this assumption, confocal laser scanning microscopy analysis was performed using Nile red as a probe to visualise SALNs in the red channel, while no probe was used for Fe@hepa since their clusters appeared as dark spots by observation in white-light channel. Observing the cells treated with Fe@hepa, the dark spots attributed to the Fe@hepa clusters seem to be localised in a different z-thickness compared to nuclei, indicating that they were not internalised by CaCo-2 cells (Figure 7A). This observation indicated that iron clusters had not entered the cells, giving evidence of the overestimation of Fe@hepa associated with the cells. On the contrary, both unloaded SALNs and SALNs–Fe@hepa5 seem to be internalised in the cells because a slight red fluorescence is noticeable around the nuclei, highlighting that the particles were localised in the cytoplasm. However, the iron particles embedded in the SALNs were not visible, probably owing to their small dimension even thought they could be considered responsible of the grey shading visible around the nuclei (Figure 8). On the other hand, regarding the black spots visible outside the cells in the image of cells incubated with SALNs–Fe@hepa, they could be attributable to Fe@hepa not embedded in the lipid matrix but only absorbed on the surface or highly dispersed in the suspension according to what was observed in the in vitro stability studies (Figure 7).

## 4. Materials and Methods

### 4.1. Chemicals

Geleol™ (Glycerol Monostearate 40–55, type I) and Gelucire 50/13^®^ Pellets (Stearoyl Macrogol-32 Glycerides) were a kind gift from Gattefossè (Saint-Priest, France). Fe@hepa (containing 9% *w*/*w* of heparin) were provided by the laboratory of Prof. E. Vismara and synthesised as previously described [20]. Azure II and Nile red were purchased from Sigma-Aldrich Italia (Milan, Italy). Potassium thiocyanate was purchased from Carlo Erba Reagenti (Milan, Italy). Hoechst 33342 stain was purchased from ThermoFisher (Monza, Italy). Dulbecco’s Modified Eagle’s Medium with high glucose (DMEM), l-glutamine, fetal bovine serum (FBS), penicillin–streptomycin (P/S), phosphate buffer saline (PBS), sodium pyruvate and other culture reagents were purchased from EuroClone (Milan, Italy).

A MilliQ water system (Millipore; Bedford, MA, USA) provided high purity water (18.2 MΩ) for these experiments.

All other chemical reagents were obtained commercially as reagent-grade products.

### 4.2. SALN Formulation

**Figure 9:**
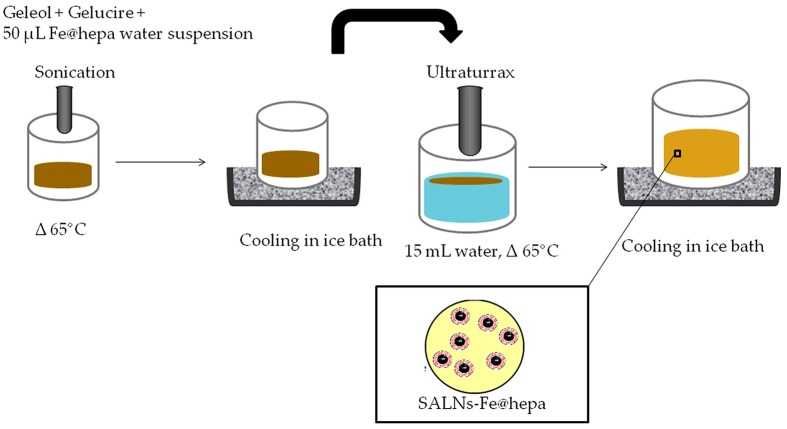
Scheme of self-assembling process used for SALN formulation.

SALNs were obtained by a self-assembling process using an original technique in which no organic solvents were employed (Figure 9). Briefly, a mixture of Geleol:Gelucire 1:1 *w*/*w* was melted at 65 °C after the addition of 50 µL of MilliQ water containing Fe@hepa (1 or 5 mg) and emulsified by ultrasound energy (Vibra-Cell, Sonic & Materials, Inc. 53 Church Hill Road, Newton, CT, USA) at 10 output Watt for 30 s. This water/oil (W/O) dispersion was rapidly solidified in ice bath and then added to 15 mL of MilliQ water, previously heated at 65 °C. To obtain the SALNs, the dispersion was homogenized for 3 min at 24,000 rpm by Ultra Turrax (T-25 basic IkaLabortecnik, Staufen, Germany) and then cooled in ice for about 10 min to allow the SALN solidification. SALNs–Fe@hepa were purified by centrifugation at 2000 rpm for 5 min at 20 °C (Rotina 380R, Hettich, Kirchlengern, Germany) to remove the non-encapsulated Fe@hepa. The purified suspensions of SALNs were stored at +4 °C. Unloaded SALNs were obtained omitting the addition of Fe@hepa in the 50 µL of MilliQ water, while labelled SALNs for cell internalisation studies were obtained by adding Nile red (0.01%) in the melted Geleol™.

### 4.3. SALN Characterisation

SALN size and polydispersity index (PDI) were determined by photon correlation spectroscopy (PCS) technique using a Zetasizer Nano ZS analyser system (Zetasizer version 6.12; Malvern Instruments, Worcs, UK). The results were expressed as the average of three different measurements.

Particle surface charge (Z-Potential value) was measured by using the same apparatus, equipped with a 4 mW He–Ne laser (633 nm) and DTS software (Version 5.0, Malvern Instruments, Worcs, UK). Measurements were performed in triplicate and each measurement was averaged over at least 12 runs.

### 4.4. Morphological Studies

SALN morphological features were analysed by field-emission gun scanning electron microscopy (SEM-FEG, Nova 11 NanoSEM 450, Fei, Eindhoven, The Netherlands) using both the SEM and the TEM mode. For the SEM mode, a few drops of the SALNs suspension were placed on an aluminum stub (TAAB Laboratories Equipment Ltd., Aldermaston, Berks, UK) covered by a double side sticky tab (TAAB Laboratories Equipment Ltd., Aldermaston, Berks, UK) and, after drying, vacuum coated with gold–palladium in an argon atmosphere for 60 s (Sputter Coater Emitech K550, Emitech LTD, Ashford, Kent, UK).

For the TEM mode a STEM detector characterised by a low voltage electron beam (30 kV) was employed. TEM 200 mesh Formvar/Carbor Coppergrids (TAAB Laboratories Equipment Ltd., Berks, UK) were immersed in SALNs diluted suspension (1:10 *v*/*v* in water) and dried at room conditions (25 °C, 760 mmHg) before the analysis.

Elemental composition of Fe@hepa loaded or unloaded SALNs was determined by energy disperse X-ray (EDX) analysis with X-EDS Bruker QUANTAX-200 (Bruker Nano GmbH, Berlin, Germany) coupled with SEM-FEG. Elements can be identified qualitatively and semi-quantitatively as a function of the X-ray energy emitted by their electrons transferring from a higher energy shell to a lower energy one. The X-ray emissions from the Kα or Lα levels were measured for the following atoms: oxygen (Kα = 0.525 keV), carbon (Kα = 0.277 keV), silicon (Kα = 1.740 keV), aluminium (Kα = 1.487 keV) and iron (Kα = 6.404 keV, Lα = 0.705 keV).

### 4.5. Drug Loading and Encapsulation Efficiency

The determination of Fe@hepa loaded in SALNs was performed by indirect method analysing the amount of non-encapsulated Fe@hepa by a spectrophotometric method based on the formation of highly-coloured complexes iron-thiocyanate ion.

Briefly, after obtaining SALNs, the separation of the non-encapsulated Fe@hepa (free Fe@hepa) was carried out by centrifugation (see Section 4.2). The pellet was re-suspended in 200 µL of milliQ water and digested in 1 mL of HCl 37% *w*/*w* for 2 h at 60 °C. Subsequently, 1.5 mL of 0.1 M solution of potassium thiocyanate (KSCN) was added to form the red-coloured [FeKSCN]^2+^ iron complex. The amount of free Fe@hepa was determined by recording absorbance at 480 nm (Lambda 35 UV/VIS, Perkin-Elmer, Norwalk, CT, USA). The standard calibration curve for iron complex was performed under identical conditions with known amounts of naked Fe@hepa and using KSCN and HCl solution as blank [38].

The encapsulation efficiency (EE%) and drug loading (DL µg/mg) were calculated by using the following equations:

$$\mathrm{EE}\%=\frac{\mathrm{initial}\;\mathrm{Fe}@\mathrm{hepa}\left(\mathrm{mg}\right)-\mathrm{free}\;\mathrm{Fe}@\mathrm{hepa}\;\mathrm{determined}\left(\mathrm{mg}\right)}{\mathrm{initial}\;\mathrm{Fe}@\mathrm{hepa}\left(\mathrm{mg}\right)}\times 100$$

$$\mathrm{DL}\left(\frac{µg}{\mathrm{mg}}\right)=\frac{\mathrm{encapsulated}\;\mathrm{Fe}@\mathrm{hepa}\left(µg\right)}{\mathrm{total}\;\mathrm{mass}\;\mathrm{of}\;\mathrm{SALNs}\;\mathrm{composition}\left(\mathrm{mg}\right)\left(\mathrm{lipids}\;\mathrm{and}\;\mathrm{Fe}@\mathrm{hepa}\right)}$$

where encapsulated Fe@hepa were calculated by subtracting the amount of free Fe@hepa determined from the initial amount added to the preparation. The experiments were conducted in triplicate and the results were expressed as average ± standard deviation (SD).

### 4.6. In Vitro SALNs-Fe@hepa Stability

In order to evaluate the in vitro stability of SALNs–Fe@hepa, the amount of Fe@hepa separated gradually at 4 °C for one month by spontaneous precipitation was quantified.

Practically at predetermined intervals (2, 4, 6, 8, 10, 20 and 30 days), the pellet deposited at the bottom of the vials of the SALNs-Fe@hepa suspension was determined. The pellet was recovered, re-suspended in 200 µL of milliQ water and digested in 1 mL of HCl 37% *w*/*w* for 2 h at 60 °C. After that, the solution was analysed to determine the amount of iron in accordance with the method described above. The analyses were performed in triplicate.

### 4.7. Stability of Heparin Coating

In order to analyse the stability of the heparin coating in Fe@hepa before and after the formation of SALNs, the amount of heparin released in saline solution from naked Fe@hepa and SALNs–Fe@hepa was evaluated.

Briefly, a known amount of sample was incubated under magnetic stirring in 0.9% NaCl water solution at 37 °C for 1 h. Then, the suspension was centrifuged for 25 min at 9500 rpm at room temperature (Rotina 380R) in order to separate nanoparticles (naked Fe@hepa or SALNs–Fe@hepa) from the supernatant. The amount of heparin released in the supernatant was determined by using a modified Azure II colorimetric method [39]. Typically, aliquots (500 µL) of aqueous solution were reacted with 4.5 mL of the Azure solution (0.01 mg/mL) and assayed at 654 nm by vis-spectroscopy (Lambda 35 UV/VIS). Quantification was achieved by comparing the absorbance of the samples to a regression curve determined from medium spiked with increasing amount of heparin. The experiments were conducted in triplicate.

### 4.8. In Vitro Nile Red Release

Nile red released from SALNs was evaluated during 24 h. Labelled SALNs (40 mg) were incubated at 37 °C in 40 mL of phosphate buffer (20 mM, pH 7.4) or DMEM added with serum, under magnetic stirring. One millilitre of SALNs suspension was withdrawn from the system at time intervals of 30 min and replaced with 1 ml of fresh solvent to maintain constant volume. The sample was centrifuged at 9500 rpm for 25 min and Nile red content was determined in the supernatant by vis-spectroscopy at 525 nm. The analysis was performed in triplicate.

### 4.9. Cell Culture

CaCo-2 cell line were cultured as a monolayer in Dulbecco’s Modified Eagle’s Medium with high glucose (DMEM) containing l-glutamine 2 mM, penicillin 100 UI/mL, streptomycin 100 µg/mL, sodium pyruvate and 10% of fetal bovine serum (FBS) at 37 °C in a humidified atmosphere (5% CO_2_). Cells were sub-cultured when the confluence was ≥80%.

### 4.10. Cytotoxicity Assay

CaCo-2 cells were seeded at a density of 60,000 cells/well in 24-well plate in complete DMEM medium for 48 h. Cells were then treated with Fe@hepa, unloaded SALNs and SALNs–Fe@hepa samples at different concentrations (0.8, 1.2, 1.6 and 2 mg/mL for SALNs, and respective Fe@hepa concentrations) for 2, 4, and 6 h.

After incubation times the methyl thiazole tetrazolium test (MTT) was performed to assess cell viability. Optical densities were measured spectrophotometrically at 570 nm with a multiplate reader (TecanGenios Pro with Magellan 6 software). Cell viability was expressed as a percentage of cell survival respect to the control (untreated cells).

The experiment was performed in triplicate.

### 4.11. Quantification of SALNs–Fe@hepa on CaCo-2 Cell Model

The amount of Fe@hepa up-taken by the CaCo-2 cells after treatment with Fe@hepa and SALNs–Fe@hepa at different incubation times was quantified by adapting a method previously reported [40]. Cells were seeded in 6-well plate at density of 250,000 cells per well in complete DMEM medium for 48 h. Then, cells were incubated with 2 mg/mL of SALNs–Fe@hepa and a proportional amount of naked Fe@hepa (53 µg/mL) for 2, 4 and 6 h. At the end of the incubation time, cells were washed with PBS and the amount of iron associated to the cells was quantified using the method described in Section 4.5. The experiments were performed in triplicate. In order to compensate the matrix effect, the calibration curve for iron quantification was prepared incubating different known amounts of Fe@hepa in the presence of unloaded SALNs into CaCo-2 cells.

### 4.12. CLSM Studies of Monolayers

The confocal laser scanning microscopy (CLSM) of fixed cells was performed with a Leica DM IRE2 microscope (Mannheim, Germany) and a Leica Confocal System equipped with a scanner multiband 3-channel Leica TCS SP2 with AOBS, laser diode blue COH (405 nm/25 mW), laser Ar (458 nm/5 mW) (476 nm/5 mW) (488 nm/20 mW) (496 nm/5 mW) (514 nm/20 mW), laser HeNe (543 nm/1.2 mW), laser HeNe (594 nm) (orange) and laser HeNe (633 nm/102 mW). CaCo-2 cells, seeded at a density of 100,000 cells per well in a chambered coverglass (Lab-Tek^®^, Thermo-scientific, Milan, Italy), were incubated with naked Fe@hepa (53 µg/mL), Nile red-labelled unloaded SALNs (2 mg/mL), and Nile red-labelled SALN–Fe@hepa (2 mg/mL). After 4 h of incubation, the treated cells were washed twice with PBS, fixed in paraformaldehyde (3% *w*/*v*) for 20 min at room temperature, stained with 2 µg/mL Hoechst 33342 dye and analysed with CLSM.

### 4.13. Statistical Analysis

Statistical analysis was performed using the one-way analysis of variance (ANOVA). The data are represented as mean ± SD. Difference was considered statistically significant at *p*-values less than 0.05.

## 5. Conclusions

In this work it was demonstrated that SALNs are an efficient carrier for Fe@hepa, reducing their cytoxicity to CaCo-2 cells and overcoming the loss of heparin coating in biological fluids. SALNs–Fe@hepa resulted able to be efficiently internalised in CaCo-2 cells, and were demonstrated to be a promising tool for delivering the theranostic Fe@hepa to lymphatic circulation by the oral route, although further studies are needed to comprehend the potential in vivo applications. Moreover, it would be interesting in the future to replace native heparin with low-molecular-weight heparins, which showed a less anticoagulant activity while maintaining antiangiogenic activity, in order to reduce risks of bleeding.

## Figures and Tables

**Table 1:** Size, polydispersity index (PDI) and Z-potential values of loaded and unloaded self-assembled lipid nanoparticles (SALNs). Fe@hepa1: iron oxide nanoparticles non-covalently coated with heparin (1 mg); Fe@hepa5: iron oxide nanoparticles non-covalently coated with heparin (5 mg).

Sample	Size (nm)	PDI	Z-Potential (mV)
Unloaded SALNs	182 ± 15	0.295 ± 0.015	−16.4 ± 4.7
SALNs–Fe@hepa1	183 ± 18	0.278 ± 0.008	−15.5 ± 5.8
SALNs–Fe@hepa5	186 ± 21	0.364 ± 0.013	−24.0 ± 5.5

**Table 2:** Encapsulation efficiency (EE%) and drug loading (DL) of SALNs–Fe@hepa.

Sample	EE%	DL (μg Fe@hepa/mg SALNs-Fe@hepa)
SALNs–Fe@hepa1	86.6 ± 2.76	5.14 ± 0.01
SALNs–Fe@hepa5	91.5 ± 3.09	26.38 ± 0.70

**Table 3:** Evaluation of the amount of heparin released in saline solution from SALNs–Fe@hepa5 and naked Fe@hepa.

Sample	Mass of Fe@hepa (μg)	Initial Amount of Heparin in Fe@hepa (μg)	% of Heparin Released in NaCl 0.9%
SALNs–Fe@hepa5	810	72.9	0
Fe@hepa	620	55.8	72
